# MRI Evaluation of Liver Iron Concentration in Patients with β-Thalassemia Major

**Published:** 2011-06-01

**Authors:** M Karimi, Vahid Emad Marvasti, Alireza Rasekhi, Perikala Vigayananda Kumar, Mohammadreza Bordbar, Alireza Moshiri, Peyman Hasanpour, Kazem Serajzadeh

**Affiliations:** 1 Hematology Research Center, Shiraz University of Medical Sciences, Shiraz, Iran; 2 Department of Radiology, Shiraz University of Medical Sciences, Shiraz, Iran; 3 Department of Pathology, Shiraz University of Medical Sciences, Shiraz, Iran

ß-thalassemia major is a hereditary anemia, characterized by a genetic deficiency in the synthesis of the ß-globin chain [1]. The main complication of the multiple blood transfusions to these patients is iron overload and the deposition of iron in various organs, such as the reticuloendothelial system, the liver, the heart and the endocrine glands [2]. Chelators also have various side effects on multiple organs in thalassemic patients, besides iron overload. Deferoxamin (DFO) which is the chelating agent most widely used for the last 30 years, mainly affects the optic, auditory and skeletal systems [3]. The effective management of thalassemic patients, especially children, requires monitoring of the toxic effects of iron overload versus chelation therapy. The most reliable method of calculating body iron stores is the biochemical or histochemical assessment of iron in a liver biopsy specimen. Since liver biopsy is an invasive procedure, attempts have been made to use imaging for detection and quantification of liver iron content [4][5][6][7]. Magnetic resonance imaging (MRI) is a technique for the diagnosis of hemochromatosis. Many investigators have shown that hepatic T2 relaxation or intensity ratios in MR images have a significant correlation with hepatic iron content [8][9][10][11]. In this study, we have evaluated the correlation of the hemochromatosis grades in liver MRIs and liver biopsies to evaluate the precision of liver MRIs in grading hemochromatosis. This double-blind cross-sectional study was conducted between January 2007 and April 2008 at the Hematology Research Center, Shiraz University of Medical Sciences. Twenty-eight blood transfusion-dependent thalassemia major patients were involved in this double-blind cross-sectional study. Thalassemia major was determined in these patients by a complete blood count (CBC) and hemoglobin electrophoresis. Patients were selected from a population that had had liver biopsies performed on them for various purposes. The exclusion criteria for this study were acute or chronic viral hepatitis and any kind of infection found in physical examinations. After obtaining the patients' written informed consent, liver MRIs (Philips 1.5 T-gyro scan, Netherlands) were performed on the patients, and T1 and T2 signal intensity in the liver was assessed at 3 points. The average of the intensities was assigned as the mean liver signal intensity of the patient. These results were compared with the results of the control group, which were MR images of 4 non-thalassemic healthy subjects with normal liver function tests by an expert radiologist who was blinded to the pathologic assessments of the patients. Liver biopsy slides were evaluated by an expert pathologist who was blinded to the study, and were graded into categories of mild, moderate and severe hemochromatosis. Histopathologic evaluation of liver biopsy slides by semiquantitative assessment of stored tissue iron revealed mild hemochromatosis in 1 patient (3.5%), moderate hemochromatosis in 11 patients (39.2%) and severe hemochromatosis in 16 patients (57.1%). The results of MRI evaluation and comparison of liver signal intensities in the patients and the control group revealed 4 (14.2%) mild, 7 (25.0%) moderate and 17 (60.8%) severe hemochromatosis. Spearman's rho test confirmed the significant correlation of MRI and liver biopsy evaluation of hemochromatosis (P < 0.001). In 22 of 28 patients (78.5%), the MRI and liver biopsy results were in agreement with each other. In 6 other cases (21.5%), the grade of hemochromatosis was higher on liver biopsy evaluation. There was also a strong correlation between the grading of hemochromatosis by MRI and by serum ferritin level (P = 0.000). Considering the fact that liver biopsy is very invasive, and that many patients refuse to submit to this procedure, we have shown that grading of liver hemochromatosis by MRI is significantly correlated with grading of hemochromatosis by liver biopsy (P < 0.001). Comparing the histopathologic and radiologic results showed that an MRI is more accurate for the diagnosis and grading of liver iron deposition in severe hemochromatosis patients.There is also strong agreement between serum ferritin and the grading of the liver by MRI. Although serum ferritin is not a suitable marker for assessing hemochromatosis, and multiple factors like infection and inflammation affect it, we have found a strong correlation between serum ferritin and the grading of hemochromatosis by MRI. This means that in place of an invasive procedure like a liver biopsy, an MRI can be useful in monitoring iron overload in thalassemia major patients. More studies with larger sample sizes are needed to support our results. However, these results are in agreement with the results of previous studies [4][5][6][7][12].
